# Co‐pathologies and biological processes beyond amyloid‐beta and tau in people with Alzheimer's disease: Evidence from clinical cohort studies

**DOI:** 10.1111/joim.70096

**Published:** 2026-04-16

**Authors:** Daniel Ferreira, Tomasz Chmiela, Maria Eriksdotter, Clifford R. Jack, Grégoria Kalpouzos, Anna Marseglia, Patrizia Mecocci, Agneta Nordberg, Dorota Religa, Eric Westman

**Affiliations:** ^1^ Division of Clinical Geriatrics, Center for Alzheimer Research, Department of Neurobiology Care Sciences and Society Karolinska Institutet Stockholm Sweden; ^2^ Facultad de Ciencias de la Salud, Las Palmas Universidad Fernando Pessoa Canarias Santa María de Guía España; ^3^ Department of Radiology Mayo Clinic Rochester Minnesota USA; ^4^ Department of Neurology Mayo Clinic Florida Jacksonville Florida USA; ^5^ Department of Neurology, Faculty of Medical Sciences Medical University of Silesia Katowice Poland; ^6^ Theme Inflammation and Aging Karolinska University Hospital Huddinge Sweden; ^7^ Aging Research Center, Center for Alzheimer Research, Department of Neurobiology, Care Sciences and Society Karolinska Institutet and Stockholm University Stockholm Sweden; ^8^ Section of Gerontology and Geriatrics, Department of Medicine and Surgery University of Perugia Perugia Italy; ^9^ Ageing Epidemiology Research Unit, School of Public Health Imperial College London London UK

**Keywords:** cholinergic, iron, neuroinflammation, senescence, synucleinopathy, TDP‐43, vascular

## Abstract

Alzheimer's disease (AD) is neuropathologically defined by amyloid‐beta (Aβ) plaques and tau neurofibrillary tangles. However, co‐pathologies and other pathobiological processes are involved in the pathogenesis of AD, contributing to neurodegeneration and clinical symptoms. The most common co‐pathologies in people with AD are alpha‐synucleinopathy, vascular brain injury and transactive response DNA‐binding protein of 43 kDa‐related pathology. Neuroinflammation, iron accumulation, cholinergic dysfunction and cellular senescence are recognized pathobiological processes beyond Aβ‐ and tau‐related pathology. However, the exact mechanisms by which these co‐pathologies and pathobiological processes contribute to the neurodegeneration and clinical symptoms in people with AD remain unclear. The individual combination of these co‐pathologies and pathobiological processes increases phenotypical heterogeneity in people with AD. This highlights the unmet need to advance their current understanding, and the field strives to develop accurate biomarkers for personalized assessment and investigation. Elucidating this biologic‐clinical complexity and heterogeneity is crucial for increasing our current understanding of AD, with implications for diagnosis, prognosis and therapeutics.

## Graphical abstract

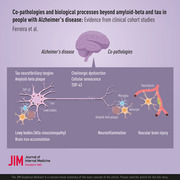

## Introduction

**Fig. 1:**
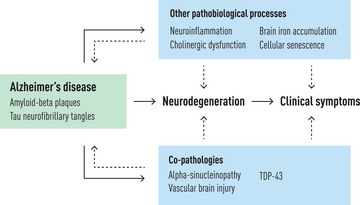
Co‐pathologies and key pathobiological processes contributing to the neurodegeneration and clinical symptoms in Alzheimer's disease (AD). AD is neuropathologically defined by amyloid‐beta plaques and tau neurofibrillary tangles. These have a direct contribution to the neurodegeneration and clinical symptoms in people with AD. However, co‐pathologies and other pathobiological processes are involved in the pathogenesis of AD, additionally contributing to the neurodegeneration and clinical symptoms. The exact mechanisms by which these co‐pathologies and pathobiological processes contribute to the neurodegeneration and clinical symptoms in people with concurrent AD remain unclear. This is reflected in the figure. Solid lines represent well‐established direct contributions, whereas dashed lines represent additional relationships that may or may not be fully supported by the current body of research. Furthermore, dashed lines may also suggest the possibility of bidirectional relationships (for example, between amyloid‐beta plaques and brain iron accumulation); however, they do not imply causality (brain iron accumulation does not cause the formation of amyloid‐beta plaques and vice versa). TDP‐43, transactive response DNA‐binding protein of 43 kDa.

Alzheimer's disease is neuropathologically defined by amyloid‐beta (Aβ) plaques and tau neurofibrillary tangles (NFT). These neuropathologic changes emerge early in the course of the disease, leading to neurodegeneration and clinical symptoms (Fig. 1). However, other pathobiological processes and additional pathologies may co‐occur and be involved in the pathogenesis of AD. The revised criteria for the diagnosis and staging of AD incorporate new biomarker categories beyond Aβ and tau, such as neuroinflammation, as well as coding for non‐AD pathologies, including alpha‐synucleinopathy and vascular brain injury, which—although being distinct diseases—frequently co‐occur in people with AD [1]. TDP‐43 (transactive response DNA‐binding protein of 43 kDa) is another common co‐pathology in AD that is not yet included in the revised diagnostic criteria of AD due to the current lack of biomarkers.

Alpha‐synucleinopathy, vascular brain injury and TDP‐43 influence neurodegeneration and clinical progression in people with concurrent AD [2] (Fig. 1). TDP‐43 and hypertensive arteriopathy may contribute to the neurodegeneration of medial temporal lobes and the development of a typical amnestic cognitive phenotype. In contrast, alpha‐synucleinopathy and cerebral amyloid angiopathy may contribute to the neurodegeneration of cortical areas and the development of more atypical cognitive phenotypes, often called non‐amnestic [2].

Neuroinflammation and other related pathobiological processes, such as iron accumulation, cholinergic dysfunction and cellular senescence (Fig. 1), have received major attention in recent years. Both neuroinflammation and iron‐related processes may precede Aβ plaque formation or be early downstream processes triggered by pre‐plaque soluble Aβ [3, 4]. Neuroinflammation could also be a later phenomenon as a response to formed Aβ plaques or, later still, as a response to neurodegeneration. The cholinergic system and the neurotrophin nerve growth factor (NGF) enhance anti‐inflammatory pathways and contribute to reducing pathologic Aβ formation [5], whereas cellular senescence exacerbates the formation of Aβ plaques and tau NFTs through the secretion of pro‐inflammatory molecules [6, 7]. These findings highlight the need to continue elucidating mechanisms in AD beyond Aβ and tau.

This perspective article reviews how co‐pathologies and several key pathobiological processes contribute to pathogenesis, neurodegeneration and clinical symptoms in people with concurrent AD. This perspective primarily relies on clinical cohort studies and aims to offer insights beyond Aβ‐ and tau‐related mechanisms.

## The contribution of co‐pathologies

### Alpha‐synucleinopathy in concurrent AD

Alpha‐synuclein is a protein encoded by the *SNCA* gene. It is mainly found in presynaptic terminals of neurons and has a central function in synaptic vesicle trafficking and neurotransmitter release. Synucleinopathy is the abnormal accumulation of insoluble forms of alpha‐synuclein in neurons, nerve fibres and glial cells, forming the so‐called Lewy bodies and neurites. The spread of synucleinopathy in the brain has been proposed to start in the brainstem (brainstem stage) or olfactory bulbs, later involving the amygdala (limbic/transitional stage) and eventually the cortex (neocortical stage) [8]. This model has been revisited, and two staging systems have recently been proposed [9, 10].

Post‐mortem studies show that 20%–65% of people with sporadic AD have concomitant synucleinopathy at autopsy [11, 12]. In autosomal‐dominant AD (ADAD), there is a substantial variability, but synucleinopathy has been reported to range from 14% to 85% [12]. Synucleinopathy has an additive effect on the clinical phenotype of people with AD, leading to a faster cognitive decline. This effect is more prominent in people with AD and neocortical synucleinopathy than people with AD and limbic/transitional synucleinopathy [12]. Some studies reported an effect of concomitant synucleinopathy on motor function, hallucinations, behaviour and sleep disturbances in people with AD [12].

The recent development of alpha‐synuclein seed amplification assays (SAA) has revolutionized the ability to assess synucleinopathy in vivo using different body samples such as cerebrospinal fluid (CSF) [13]. The use of alpha‐synuclein SAA in AD clinical studies has accelerated considerably during the last 3 years, although the literature is still limited to a few cohorts, including Alzheimer's Disease Neuroimaging Initiative (ADNI) and local cohorts in Sweden, Italy, Germany and France. The first alpha‐synuclein SAA publication is from 2023, which showed that 45% of people with AD dementia had a positive alpha‐synuclein SAA and presented with a higher frequency of atypical phenotypes, parkinsonism and orthostatic hypotension than those counterparts with a negative alpha‐synuclein SAA [14]. Later studies have reported lower frequencies of a positive alpha‐synuclein SAA in people with sporadic AD dementia, with numbers ranging from 14% to 39% [15, 16, 17, 18]. This frequency may be lower in symptomatic ADAD, with a study reporting 11% [19]. The frequency of a positive alpha‐synuclein SAA in AD increases with age and clinical severity, with a frequency of 0% in asymptomatic ADAD, 12%–27% in preclinical sporadic AD and 17%–26% in prodromal sporadic AD [15, 16, 18, 19, 20].

A positive alpha‐synuclein SAA in individuals along the continuum of AD is associated with greater cognitive impairment, presence of behavioural disturbances and an increased risk for future cognitive decline, independent of AD pathology [15, 16, 18, 20, 21]. A positive alpha‐synuclein SAA also influences neurodegeneration. For example, four concurrent studies from the ADNI cohort showed that a positive alpha‐synuclein SAA is associated with greater hypometabolism as assessed with ^[18F]^fluorodeoxyglucose positron emission tomography (FDG‐PET), particularly in the occipital cortex [22, 23, 24, 25]. Interestingly, people with AD and a positive alpha‐synuclein SAA had hypometabolism disproportionate to atrophy on magnetic resonance imaging (MRI) or tau PET binding [25]. This finding was independent of vascular and TDP‐43 co‐pathologies but was concurrent to lower CSF levels of dopamine metabolites and synaptic markers. The authors suggested that altered neurotransmission and neuron integrity may contribute to the dissociation between hypometabolism in occipital cortex and atrophy and tau PET binding in people with AD and a positive alpha‐synuclein SAA [25]. Another study showed that a positive alpha‐synuclein SAA is associated with reduced volume of nucleus basalis of Meynert (nbM), independent of AD pathology. As for FDG‐PET, nbM mediated the effect of synucleinopathy on cognitive performance, independent of AD pathology [26].

An interesting line of research is the interplay between Aβ, tau and synucleinopathy biomarkers in vivo. A positive alpha‐synuclein SAA may be more likely in the presence of Aβ positivity but not tau positivity [16, 20]. However, a study showed that alpha‐synuclein SAA conversion from negative to positive is not associated with Aβ and does not impact progression of AD pathology [21]. In ADAD, alpha‐synuclein SAA may become positive downstream to AD pathology [19].

Despite intense research, there is no reliable PET tracer for neuronal synucleinopathy. However, a recent study suggests that the ^[18F]^ACI‐12589 tracer may be relevant for glial alpha‐synuclein, which is characteristic of multiple system atrophy [27]. Developing a PET tracer for neuronal synucleinopathy is a high priority in the field. Two other priorities are the development of blood‐based alpha‐synuclein biomarkers and the clinical implementation of alpha‐synuclein SAA. The clinical implementation of alpha‐synuclein SAA requires harmonization of protocols across centres, consensus on definition of a positive SAA test result and developing an international quality assurance scheme. The VALID study addressed some of these challenges, demonstrating variation in the performance of alpha‐synuclein SAA across four European centres [28].

#### Vascular brain injury in concurrent AD

Vascular brain injury or vascular pathologies are the hallmarks of cerebrovascular disease (CVD), a condition caused by impairment of blood flow and structure and function of cerebral blood vessels [29]. Large vessel disease is typically caused by atherosclerosis and affects major arteries, leading to stenosis, occlusion or embolism. This may result in ischaemic or embolic stroke. In contrast, small vessel disease (SVD) involves small penetrating arteries, arterioles, capillaries and venules within the brain parenchyma or subarachnoid space [29]. SVD manifests as small subcortical infarcts, lacunes, white matter hyperintensities, perivascular spaces, cerebral microbleeds, cortical superficial siderosis, cerebral microinfarcts and atrophy, which are all detectable on MRI [29].

CVD is a frequent co‐pathology in AD [1]. Post‐mortem studies show that CVD exists in 70%–90% of people with sporadic AD. Aβ can deposit in the vessel walls, compromising vascular integrity and contributing to conditions such as cerebral amyloid angiopathy and dementia in patients with concurrent AD [30]. CVD poses a critical challenge to anti‐amyloid therapies. For instance, patients with AD who are positive for certain CVD biomarkers are not eligible for monoclonal amyloid antibody treatments due to a theoretical increased risk for amyloid‐related imaging abnormalities (ARIA; brain swelling and/or bleeding) [31].

The mechanisms by which CVD contribute to neurodegeneration in people with concurrent AD are multifactorial. Chronic cerebral hypoperfusion induces neuronal energy deficits, oxidative stress and hypoxia [32]. Blood–brain barrier breakdown facilitates entry of neurotoxic and inflammatory agents into the brain, accelerating neuronal injury [32]. Vascular dysfunction could possibly impair clearance of Aβ, other proteinopathies and neurotoxins, thereby promoting their accumulation in the brain [33]. Impaired clearance through glymphatic secretion reduces amyloid‐β removal, a process critically dependent on the perivascular polarization of aquaporin‐4 water channels on astrocytic endfeet [34]. SVD further exacerbates this dysfunction by damaging perivascular spaces and vascular integrity, which are essential for efficient glymphatic transport [35]. Additionally, CVD can trigger neuroinflammation, which in turn impairs aquaporin‐4 localization and may exacerbate tau pathology and Aβ plaque spreading [36]. SVD has also been linked to both neuroinflammation and insulin‐related dysregulations [37, 38], which are biological factors implicated in AD pathogenesis [39]. Yet, the links between CVD, AD pathogenesis and progression, and cognitive impairment and other clinical symptoms are not fully known.

Clinically, CVD may lower the threshold for the clinical manifestation of dementia. A recent study highlighted that SVD can exacerbate the clinical symptoms in people with a low AD pathological burden [40]. Yet, whether CVD and AD act additively or synergistically remains unclear and warrants further research. This complexity is compounded by the heterogeneity in severity and location of the vascular brain injury [41], which may also contribute to variability in the presentation of clinical symptoms in people with concurrent AD [42]. Neuroimaging studies using grey matter (MRI), glucose metabolism (FDG‐PET) and tau PET uptake patterns have consistently identified four subtypes of AD. These AD subtypes show distinct rates of cortical atrophy influenced by vascular factors [43]. One study found that hypertensive arteriopathy and cerebral amyloid angiopathy are vascular co‐pathologies associated with specific AD subtypes [2]. Furthermore, white matter hyperintensities contribute to the neurodegeneration in AD, for example, affecting the cholinergic system at early stages of the disease [44].

Smoking, poor diet, physical inactivity, obesity, hypertension, hypercholesterolaemia and diabetes are modifiable risk factors of CVD [41, 45]. These risk factors can also be present in people with AD, but whether they can influence Aβ plaque and tau NFT formation is unclear. Furthermore, although both AD and CVD contribute to the neurodegeneration [46], individuals age differently due to a combination of individual risk factors and resilience mechanisms. These resilience mechanisms help mitigate the accumulation of pathology and slow down the rate of cognitive decline [46]. However, the brain's capacity to tolerate AD and co‐pathologies remains poorly understood.

#### TDP‐43 in concurrent AD

TDP‐43 stands for transactive response DNA‐binding protein of 43 kDa. The TDP‐43 is encoded by the *ARDBP* gene on chromosome 1 and is composed of 414 amino acids [47]. Its function includes RNA splicing, mRNA turnover, RNA trafficking and biogenesis of mRNA transcripts [47]. TDP‐43 can downregulate tau expression [48]. TDP‐43 pathology involves its depletion from the nucleus and transfer to the cytoplasm, where it undergoes multiple post‐translational modifications—including ubiquitination, phosphorylation and truncation—eventually leading to the formation of insoluble aggregates [47].

The pathologic forms of TDP‐43 were first discovered in 2006 in tau‐negative individuals with amyotrophic lateral sclerosis (ALS) and frontotemporal lobar degeneration (FTLD). Most forms of ALS and FTLD are characterized by cytoplasmic inclusion of TDP‐43 [47]. However, TDP‐43 has also been identified in 30%–70% of people with concurrent AD [47, 49]. In addition, TDP‐43 is found in the aged brain, which has been termed limbic‐predominant age‐related TDP‐43 encephalopathy [50]. The exact mechanism by which TDP‐43 may contribute to the development of AD remains unclear [47], but it is hypothesized that it may promote AD through both Aβ‐dependent and independent pathways [51]. TDP‐43 tends to colocalize with Aβ plaques and tau NFTs, and the presence of TDP‐43 is associated with advanced stages of Aβ and tau NFT pathology [52]. TDP‐43 and Aβ share structurally similar domains that could lead to the formation of amyloid‐TDP‐43 complexes, possibly as a result of the cross‐seeding capacity of both TDP‐43 and Aβ [53]. Furthermore, TDP‐43 oligomers can be recognized by an anti‐amyloid‐specific antibody [54]. In mouse models, TDP‐43 inhibits Aβ fibrillization at initial and oligomeric states through its N‐terminal domain, enhances Aβ‐induced neurotoxicity and memory deficits and exacerbates AD pathology by interacting with Aβ and promoting neuroinflammation [55]. These data further support a direct interaction between TDP‐43 and Aβ.

The possible link between TDP‐43 and tau has also been studied. Tau oligomers increase nuclear levels of both phosphorylated and non‐phosphorylated TDP‐43 monomers in a dose‐dependent manner [56], leading to TDP‐43 translocation to the cytoplasm, polymerization and aggregation. Further, tau oligomers may be able to cross‐seed with TDP‐43 [56]. In autopsies of AD brains, TDP‐43 has been found to colocalize with phosphorylated tau [57]. However, contradictory reports also exist, which showed a negative association between TDP‐43 and tau in post‐mortem AD brains, suggesting an inhibitory effect of TDP‐43 on tau transcripts [48]. This might indicate that TDP‐43 loss of function promotes tau pathology in AD.

Another potential link between TDP‐43 and AD is the apolipoprotein E gene (*APOE*). People with AD who are carriers of the *APOE* ε4 allele are more likely to have TDP‐43 co‐pathology and are characterized by more severe cognitive decline [58, 59].

Clinically, people with AD and TDP‐43 co‐pathology are characterized by more severe cognitive impairment, greater brain atrophy and, in particular, more severe hippocampal atrophy [59]. The prevalence of TDP‐43 co‐pathology differs between subtypes of AD, being more common in limbic‐predominant and typical AD subtypes—67% and 59%, respectively—than in the hippocampal‐sparing AD subtype (21%) [59]. In addition, people with late‐onset AD are characterized by higher cortical TDP‐43 pathology, which further supports the association of TDP‐43 with a more severe phenotype in people with concurrent AD [59].

## Neuroinflammation in AD

Neuroinflammation refers to different inflammatory responses induced by pathology in the brain. Neuroinflammation is characterized by production of different pro‐inflammatory molecules that can induce synaptic dysfunction and neuronal death. Innate immune glia cells, such as astrocytes and microglia, are involved in these processes. Among the neuroglia cells, astroglia are the main homeostatic cells abundant with house‐keeping functions in the neuronal milieu. Astroglia contribute to control neurotransmitter release, recycling, energy metabolism and communication with other cellular and non‐cellular components in normal and pathological brains [60, 61]. Normally, microglia maintain control of neuronal plasticity as well as phagocytic function of debris, whereas under pathological conditions microglia can cause a reduction in trophic factors, excessive synaptic pruning, and elimination of synaptic structures [62, 63].

Reactive astrocytes have been suggested as an umbrella term for astrocytes undergoing remodelling to different states, function and properties due to different pathologies [64]. The existence of multiple subtypes of astrocytes in AD has been recently demonstrated in two single‐nuclei RNA sequencing studies [65, 66]. Immunohistochemistry staining of glial fibrillary acidic protein (GFAP) has routinely been used as a marker for astrogliosis in post‐mortem brain tissue. However, GFAP antibodies most likely cannot label the whole range of brain astrocytes [61] as supported by recent transcriptomic studies [65, 66]. Astrocytes can be measured as plasma GFAP [67] and by using the monoamine oxidase B (MAO‐B) PET tracer ^[11C]^deprenyl, which has shown increased astrogliosis in Aβ positive people with sporadic prodromal AD [68]. In mutation carriers of ADAD, ^[11C]^deprenyl PET has revealed peak levels of astrogliosis 15–20 years before the expected onset of clinical symptoms. Further, ^[11C]^deprenyl PET undergoes a divergent time course pattern as the disease progresses, in contrast with the cumulative pattern of increased ^[11C]^amyloid PIB PET [3]. These recent findings suggest that reactive astrogliosis may promote Aβ plaque formation or be triggered by pre‐plaque soluble Aβ. Based upon multi‐tracer clinical/translational in vivo to in vitro PET imaging, a “Two wave model of reactive astrogliosis” in AD has been suggested [69, 70], which requires further insights into the heterogeneity in reactive astrogliosis. Furthermore, a strong negative correlation has been observed between plasma GFAP levels and astrocyte ^[11C]^deprenyl levels in both ADAD and sporadic AD, which suggests that the two biomarkers may detect different states or subtypes of astrogliosis [71].

^[18F]^SMBT‐1 [72] and ^[11C]^BU9908 [73] are two new additional MAO‐B PET tracers for measuring astrogliosis in patients with AD and Parkinson's disease. ^[18F]^SMBT‐1 was developed as a reversible MAO‐B inhibitor and ^[11C]^BU99008 as a PET tracer targeting the type 2 imidazoline receptor binding sites (I_2_‐BS) in the mitochondria. In vitro autoradiography studies in post‐mortem AD brain tissue have demonstrated that deprenyl, SMBT‐1 and BU99008 bind to the same MAO‐B binding sites in mitochondria. However, BU99008 binds to an additional independent binding site—most probably I_2_‐BS—which opens up for further studies on the complexity of reactive astrogliosis and multiple forms or subtypes of astroglia in the AD continuum [74].

As observed in post‐mortem AD brains, the assembly of glial cells around Aß plaques might occur as a response to formation of neuritic dystrophy [75]. When Aß aggregates, microglia cause Aß phagocytosis, release of pro‐inflammatory cytokines causing chronic inflammation, tau hyperphosphorylation, formation of tau NFT and neuronal death [75]. Similarly, activation of astrocytes causes release of pro‐inflammatory cytokines, which can influence microglia and the release of aggregated tau forms [76].

The in vivo visualization of microglia activation in AD requires the development of new specific PET tracers. ^[11C]^PK11195 represents the first generation of tracers targeting the translocator protein (TSPO). ^[11C]^PK11195 has been extensively used, and a series of second‐generation TSPO tracers has been developed, including ^[11C]^PBR28, ^[11C]^DA1106 and ^[11C]^DPA713. However, the binding affinity of these second‐generation TSPO tracers is unfortunately influenced by a polymorphism in the TSPO gene, and research is ongoing to find more specific microglia PET tracers.

The role of neuroinflammation is a hot topic in AD. The existing subtype heterogeneity for both astrocytes and microglia is promising for developing new specific inflammatory biomarkers and new drug targets.

## Brain iron accumulation in AD

Intra‐cellular non‐haem iron is crucial for neurobiological mechanisms such as myelination and synaptic plasticity, as well as synthesis of DNA, neurotransmitters and adenosine triphosphate in mitochondria. However, a dysregulation of iron metabolism leading to excessive labile iron (i.e., not bound to ferritin, the storage protein of iron) is deleterious to the cells. Ferroptosis, a programmed cell death, involves free (ferrous) iron entering the Fenton reaction, leading to reactive oxygen species production, further contributing to neuroinflammation and, ultimately, cell death [4].

In humans, the first studies on brain iron using histopathological methods on post‐mortem data revealed abnormal iron deposits in AD. Already in 1953, precipitates of an iron substance within the cytoplasm of the nerve cells were observed [77]. Further, an iron‐staining substance existed in large amounts in cells that had undergone neurofibrillary degeneration, within the plaques and in the microglia [77]. On the basis of these observations, it was hypothesized that the AD pathogenesis may be a primary disturbance in the cerebral metabolism of iron.

More recently, animal research has highlighted a bidirectional relationship between iron and the Aß precursor protein (APP), such that the expression of APP is regulated by iron, and iron is affected by APP. Moreover, Aß has binding sites for iron, and together they act deleteriously. Iron and tau are colocalized, and here too there seems to be a bidirectional influence, but more investigation is needed to uncover their combined contribution to neurodegeneration [4]. These findings suggest that brain iron overload is a co‐pathological feature of AD, potentially amplifying AD neuropathology.

Research on brain iron accumulation has been greatly facilitated in humans in vivo using MRI. Relaxometry (*R*2*) and quantitative susceptibility mapping, sensitive to magnetic elements in tissue, have been validated against post‐mortem data as reliable proxies of brain iron content [78, 79]. Studies show significant contribution of brain iron overload and accumulation on functional neural disturbances, brain atrophy and cognitive decline in normal ageing and people with AD [80, 81, 82, 83, 84, 85, 86, 87], and associations between brain iron overload and neuroinflammation, Aß and tau [84, 88, 89].

The combined possibility of quantifying brain iron in vivo and blocking ferroptosis has triggered clinical trials in AD targeting brain iron. However, clinical trials that used iron chelators in AD mostly reported the absence of positive outcomes, possibly due to a plethora of factors such as the inability of iron chelators to cross the blood–brain barrier, timing of intervention and patient heterogeneity [90]. The most recent clinical trial showed that chelator deferiprone reduced brain iron load but also increased atrophy and cognitive decline with respect to the placebo group [91]. Possible explanations were a high dosage of deferiprone (removing too much iron is likely deleterious) and a cascade of events leading to disturbances in other systems, such as the dopaminergic system, tightly linked with iron metabolism [82, 92]. New clinical trials may combine iron chelators, antioxidants and other drugs to remove extra iron without compromising other systems.

Identifying modifiable factors that modulate brain iron accumulation could be a way to prevent iron accumulation. A few studies have investigated health and lifestyle factors, only in normal ageing. Despite the short follow‐up time of less than 3 years, a study showed that higher levels of iron in the blood were associated with higher brain iron accumulation in cortex and basal ganglia, and, specifically in older individuals, a worsening of cardiovascular health over time was related to more brain iron accumulation [93]. Other potential factors seem promising but need to be replicated, such as diet and nutrients, alcohol intake, smoking and physical activity [93, 94]. Future intervention studies should provide more direct evidence on whether these factors can contribute to reducing brain iron accumulation in old age and to preventing AD.

## Cholinergic dysfunction in AD

Cholinergic neurons are important for memory and attention. The cholinergic cell bodies in the basal forebrain produce the neurotransmitter acetylcholine (ACh) and innervate a wide range of brain tissues, including the cortex and hippocampus. The cholinoceptive cells possess the cholinergic receptors (muscarinic and nicotinic) and can respond to available ACh. Already in the 1970s, it was shown that cognitive decline was associated with central cholinergic dysfunction and neurodegeneration of the cholinergic basal forebrain [95]. Later studies have shown that the cholinergic neurons in the basal forebrain degenerate early in AD [96], and a correlation between reduced basal forebrain volume and cognitive decline has been demonstrated [97].

This knowledge led to the development of substances with the aim to increase cholinergic signalling as a therapeutic target for AD. The cholinesterase inhibitor class of drugs (ChEIs)—which inhibits the degradation of ACh by AChE, thereby enhancing ACh availability and signalling—was developed in the 1990s and alleviates cognitive symptoms in patients with AD [98]. Meta‐analyses have shown less cognitive decline in patients treated over 1 year, as compared with the placebo group [99]. Epidemiological studies with more than 5 years of follow‐up have shown that treatment with ChEIs is associated with persistent long‐term, albeit small, cognitive benefits [100].

In patients with AD treated with ChEIs, studies have shown an increase in brain glucose metabolism on FDG‐PET [101] and less brain atrophy on MRI [102], which suggests that the ChEIs may not only have symptomatic effects. However, conventional thinking is that ChEIs may offer some symptomatic improvement but do not affect the neurodegenerative trajectory of AD.

Interestingly, mounting evidence suggests that ChEI may play an important role outside the CNS. There are significant associations between ChEI treatment and reduced risk for renal dysfunction [103] or cardiovascular diseases [104], including reducing hospitalizations for heart failure in patients with both AD and heart failure [105]. In addition, cohort studies have shown reductions in mortality rate (around 30%) [106]. These effects may be due to the known anti‐inflammatory effects of the ChEIs and further suggest that the effects of ChEIs extend beyond the cholinergic system in the brain [107].

Several factors impact the cholinergic system. One of the most important factors is the neurotrophin NGF on which the central cholinergic neurons depend for their survival and plasticity [108]. Physiologically, NGF enhances cholinergic signalling and anti‐inflammatory pathways as well as controls APP β‐processing, thereby reducing pathologic Aβ formation [5]. Impaired NGF signalling and retrograde transport from the hippocampus and cortex to basal forebrain cholinergic neurons result in inadequate cholinergic stimulation of hippocampal circuits [96, 109]. This functional NGF deprivation accelerates cholinergic degeneration and hippocampal network failure in AD [109]. This makes NGF interesting as a therapeutical substance [109, 110]. One caveat is that NGF does not pass the blood–brain barrier. Early attempts using intracerebroventricular administration of NGF to three patients with AD [111] showed great promise in alleviating AD‐related symptoms but exhibited side effects due to NGF's natural ability to induce pain [112]. However, a mutated form of human NGF named painless NGF (hNGFp), discovered in 2004 [113], has been shown to sustain the ability to enhance cholinergic signalling without eliciting pain [114]. In addition, when delivering NGF directly to the brain tissue, no pain was elicited. This has clearly been shown in subsequent trials of patients with AD using AAV (adeno‐associated virus) delivery [115] or using the encapsulated cell biodelivery method, showing positive effects on cognition and EEG, FDG‐PET [116] brain atrophy on MRI [117] and CSF cholinergic biomarkers [118]. However, the obvious invasive nature of these delivery methods made it important to look for other routes of delivery. Studies on ways to pass the blood–brain barrier—for example, by using cytochrome *c* as a transport vehicle—are underway. Other routes, such as intranasal delivery, are being investigated, as are modulators of neurotrophic receptors able to pass the blood–brain barrier [119].

In conclusion, the goal is a future combination therapy that targets the cholinergic system. ChEIs’ positive effects on both cognitive and non‐cognitive systems make them agents of interest and motivate the recommendation for their use. Data suggest that adding stimulators of the cholinergic system to anti‐amyloid therapies, in addition to recommending preventive lifestyles, has the potential to become an attractive cocktail to treat people with AD.

## Cellular senescence and AD

Cellular senescence is a biological process in which cells stop dividing in response to many stressors, including telomere shortening, DNA damage and oxidative stress. Although these cells remain metabolically active, they adopt a distinctive phenotype marked by cell cycle arrest, resistance to apoptosis and secretion of pro‐inflammatory molecules known as the senescence‐associated secretory phenotype (SASP). At the biological level, senescence is protective by preventing the proliferation of damaged cells, contributing to processes such as tumour suppression and tissue repair [120]. However, its chronic activation and accumulation—particularly during ageing—promote neuroinflammation and tissue dysfunction, driving age‐related diseases such as cancer and AD [121].

In AD, senescent cells accumulate in the brain, exerting deleterious effects through the secretion of SASP. This phenomenon exacerbates pathological hallmarks of AD, including Aβ plaques and tau NFT, further accelerating cognitive decline [6]. Various brain cell types—including neurons, microglia, astrocytes, oligodendrocyte precursor cells and Blood Brain Barrier (BBB) cells—exhibit senescence, exacerbating neurodegeneration through loss of function and release of toxic factors [122]. For instance, neurons in a state of ‘neuroquiescence’ lose functional integrity, whereas senescent microglia and astrocytes amplify inflammatory cascades [36].

Various brain cell types contribute to the senescence observed in AD. Astrocytes—vital for maintaining neuronal homeostasis—show altered functionality and increased SASP secretion upon entering senescence. These changes facilitate Aβ aggregation and tau hyperphosphorylation, which disrupt synaptic signalling and impair the BBB. Studies by Cohen and Torres [123] highlighted that senescent astrocytes play a direct role in the progression of AD pathology. Similarly, microglial cells—the brain's immune sentinels—adopt a pro‐inflammatory phenotype upon senescence. This state—termed ‘microglial dystrophy’—has been strongly associated with the exacerbation of neuroinflammation and synaptic pruning, which are hallmarks of AD.

Mitochondrial dysfunction, oxidative stress and DNA damage are key drivers of cellular senescence in AD. Oxidative stress—a prominent feature in ageing—impairs cognitive functions and neurogenesis, further amplifying the effects of AD. Moreover, the accumulation of DNA damage triggers the activation of senescence pathways—including p53 and p16INK4a—which have been identified as markers of senescent cells in AD [124].

The link between cellular senescence and AD is also evident in the context of neuroinflammation. SASP factors such as interleukin‐6 and tumour necrosis factor‐alpha create a pro‐inflammatory environment that disrupts neuronal signalling and exacerbates tau NFT pathology [7]. Moreover, senescence markers are highly expressed in post‐mortem AD brains, particularly in areas affected by Aβ deposition and tau NFT (reviewed in Zhu et al. [125]). These findings suggest a direct association between senescent cell burden and the severity of AD pathology. Childs et al. [126] demonstrated that the clearance of senescent cells in a mouse model of AD significantly reduced neuroinflammation and improved cognitive performance. This study underscores the potential of targeting senescent cells as a therapeutic strategy in AD.

Emerging therapeutic strategies—collectively termed senotherapeutics—aim to mitigate senescence‐associated damage through senolytics (e.g., dasatinib and quercetin) that clear senescent cells or senomorphics that suppress SASP effects (reviewed in Boccardi et al. [127, 128]). Early trials demonstrate promising outcomes, such as reduced AD pathology and improved cognition, though challenges persist in identifying senescence‐specific biomarkers and addressing cell‐type heterogeneity. Dasatinib and quercetin, a combination of senolytics, were reported by Xu et al. [129] to significantly reduce senescent cell burden in aged mice, reducing Aβ pathology and improving memory. Similarly, therapeutic approaches targeting SASP factors are being investigated. These findings suggest that modulating the secretory profile of senescent cells may provide an alternative therapeutic avenue for AD. The translation of senescence‐targeting therapies to human clinical trials is underway. A Phase I trial assessed the safety and feasibility of senolytic agents in people with AD [130]. Preliminary results of a Phase II trial indicate that these interventions are well‐tolerated [131], paving the way for more extensive studies. Moreover, emerging research highlights the potential of combining senescence‐targeting therapies with conventional AD treatments.

Despite promising advancements, several challenges remain in the field of senescence research. One major limitation is the identification of specific biomarkers to detect and monitor senescent cells in human brains accurately. Efforts are underway to develop imaging techniques and fluid biomarkers to address this gap. Additionally, the heterogeneity of senescent cells poses a challenge for therapeutic interventions. Senescent astrocytes, microglia and neurons exhibit distinct phenotypes, necessitating tailored approaches to target these cells effectively. Lastly, the potential side effects of senescence‐targeting therapies—including off‐target effects and toxicity—require careful evaluation. Long‐term studies are needed to assess the safety and efficacy of these interventions in diverse populations.

## Final remarks and conclusions

This article reviews the contributions of co‐pathologies and several key pathobiological processes towards pathogenesis, neurodegeneration and clinical symptoms in people with AD. We have primarily offered insights beyond Aβ‐ and tau‐related mechanisms, based on clinical cohort studies. The basic biological mechanisms underlying these, as well as the extensive findings from community‐based studies, are out of the scope of this article and are covered by two related articles [132, 133] from the symposium ‘Beyond amyloid and tau: The importance of co‐pathologies in Alzheimer's disease’ published in the *Journal of Internal Medicine*.

Table 1 summarizes the key conclusions of this article.

**Table 1:** Conclusions.

Non‐AD co‐pathologies and pathobiological processes such as neuroinflammation, iron accumulation, cholinergic dysfunction and cellular senescence contribute to the neurodegeneration and clinical symptoms in AD [3, 6, 12, 15, 16, 18, 20, 21, 22, 23, 24, 25, 26, 40, 42, 43, 44, 46, 58, 59, 75, 80, 81, 82, 83, 84, 85, 86, 87, 95, 96, 97, 109, 116, 117, 118, 122, 126]
Between 20% and 90% of people with AD have concomitant synucleinopathy, CVD and/or TDP‐43 at autopsy [11, 12, 30, 47, 49, 59]
Synucleinopathy accelerates cognitive decline in people with concurrent AD, contributes to the development of atypical symptoms and intensifies the neurodegeneration of key brain areas such as the cholinergic nucleus basalis of Meynert [12, 14, 15, 16, 18, 20, 21, 22, 23, 24, 25, 26]
TDP‐43 also contributes to cognitive impairment in people with concurrent AD, but mostly to the typical memory symptoms and intensifies the neurodegeneration of key related areas such as the hippocampus [55, 58, 59]
CVD lowers the overall threshold for the manifestation of clinical symptoms in people with concurrent AD. The clinical contributions of CVD are, however, heterogeneous because of the broad spectrum of injuries, their severity and location [2, 40, 41, 42, 43]
CVD is involved in biological mechanisms related to neuroinflammation, brain iron accumulation, cholinergic dysfunction, neuronal energy deficit and oxidative stress [32, 37, 38, 44]
Neuroinflammation processes are early events in the pathogenesis of AD and develop through longitudinal patterns divergent from those of Aβ accumulation. Neuroinflammation plays a role in Aß‐ and tau‐related pathways [3, 69, 70, 75, 76]
Brain iron accumulation is common in AD; it has bidirectional relationships with Aß, tau, cardiovascular health and neuroinflammation and contributes to the neurodegeneration via ferroptosis and to cognitive decline [4, 77, 80, 81, 82, 83, 84, 85, 86, 87, 88, 89]
Cholinergic dysfunction is a major driver of cognitive impairment in people with AD. Drugs increasing cholinergic signalling in the brain are currently the main symptomatic treatment of AD, while they have also shown a potential to slow down the neurodegenerative process [95, 96, 97, 98, 99, 100, 101, 102, 116, 117, 118]
Drugs increasing cholinergic signalling also have anti‐inflammatory effects, and their action may extend beyond the cholinergic system of the brain [103, 104, 105, 106, 107]
Senescent cells stop dividing in response to stressors, promote neuroinflammation and neurodegeneration and contribute to age‐related diseases such as AD [7, 36, 121, 122, 126]

*Note*: The table summarizes the main conclusions of this perspective article regarding the contribution of co‐pathologies and key pathobiological processes towards disease pathogenesis, neurodegeneration and clinical symptoms in people with concurrent AD. The reviewed evidence is primarily derived from clinical cohort studies and focuses on processes beyond Aβ‐ and tau‐related mechanisms.

Abbreviations: AD, Alzheimer's disease; Aβ, amyloid‐beta; CVD, cerebrovascular disease; TDP‐43, transactive response DNA‐binding protein of 43 kDa.

**Fig. 2:**
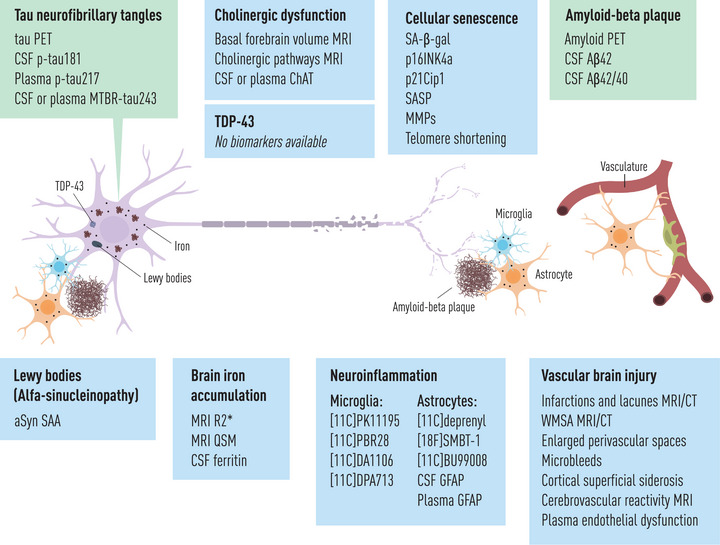
Biomarkers of Alzheimer's disease (AD), co‐pathologies and key pathobiological processes (present and future). The figure represents current biomarkers as well as biomarkers that are currently being investigated and may have the potential to be established in the future. Microtubule‐binding region (MTBR)‐tau243 in cerebrospinal fluid (CSF) or plasma is a recently developed tau assay that is more closely linked with the onset of abnormal tau positron emission tomography (PET) and correlates better with tau PET than with amyloid PET. Choline acetyltransferase (ChAT) is the enzyme that synthesizes acetylcholine, and its activity can be measured in the CSF or plasma. Relaxometry (R2*) and quantitative susceptibility mapping (QSM) are magnetic resonance imaging (MRI) sequences sensitive to magnetic elements in tissue and have been validated against post‐mortem data as reliable proxies of brain iron content. The Biomarkers for Vascular Contributions to Cognitive Impairment and Dementia (MarkVCID) consortium (https://markvcid.partners.org/) has assessed and validated some of the biomarkers for vascular brain injury listed in the figure. There are currently no biomarkers specific for transactive response DNA‐binding protein of 43 kDa (TDP‐43). Glial fibrillary acidic protein (GFAP) in the CSF or plasma is commonly considered a biomarker of astrocytic activation. Although CSF or plasma GFAP does not perfectly correlate or show the same time course than PET biomarkers of neuroinflammation, they are included in the same category for the purposes of this article. Plasma p‐tau217 is considered a biomarker of phosphorylated and secreted AD tau and is classified as a tau biomarker, although it often correlates with amyloid biomarkers in PET or CSF. Amyloid‐beta oligomers and tau fibrils precede the formation of amyloid‐beta plaques and tau neurofibrillary tangles, and they contribute to the neurodegeneration; however, they are not represented in this figure. aSyn SAA, alpha‐synuclein seed amplification assay; Aβ42, amyloid‐beta 42; CT, computed tomography; WMSA, white matter signal abnormalities.

Despite the reviewed discoveries and major scientific advances, many questions remain to be answered. Some important perspectives for the future research are listed in Table 2, and Fig. 2 represents the present and future biomarkers of co‐pathology and key pathobiological processes in people with AD.

**Table 2:** Perspectives for the future research.

The exact mechanisms by which synucleinopathy, CVD and TDP‐43 co‐pathologies contribute to AD pathogenesis, if at all, remain unclear. Future studies should consider both Aβ‐dependent and independent pathways to the neurodegeneration and clinical symptoms. The emergence of new biomarkers is envisioned to elucidate the interplay between Aβ, tau, synucleinopathy, TDP‐43 and CVD in vivo
Developing a reliable positron emission tomography tracer for neuronal synucleinopathy, a blood‐based alpha‐synuclein biomarker and implementing the alpha‐synuclein seed amplification assay clinically are high priorities
Developing positron emission tomography and fluid biomarkers for TDP‐43 are of utmost interest
New specific positron emission tomography tracers are also needed for the in vivo assessment of microglia activation in people with AD
More granular magnetic resonance imaging and fluid biomarkers of CVD and vascular brain injury are under way
Elucidating the complexity and heterogeneity of astrocyte‐ and microglia‐related processes is crucial for the identification of new drug targets
More investigation is needed to uncover the combined contribution of iron, Aß and tau towards the neurodegeneration in AD
Future studies should clarify whether targeting vascular risk factors may modulate brain iron accumulation and prevent age‐related diseases such as AD
The use of nerve growth factor not only as an enhancer of cholinergic signalling but also for reducing the neuroinflammation and pathologic Aβ formation needs to be investigated further. Brain blood barrier passage of nerve growth factor needs to be improved
The senescence field is advancing towards identifying specific biomarkers to detect and monitor senescent cells, understanding cell‐type heterogeneity and carefully evaluate potential side effects of senescence‐targeting therapies

*Note*: The table summarizes the main perspectives for the future research identified in this perspective article, with a focus on the contribution of co‐pathologies and key pathobiological processes towards disease pathogenesis, neurodegeneration and clinical symptoms in people with concurrent AD.

Abbreviations: AD, Alzheimer's disease; Aβ, amyloid‐beta; CVD, cerebrovascular disease; TDP‐43, transactive response DNA‐binding protein of 43 kDa.

Overall, the current perspective article highlights the important contributions of co‐pathologies and key pathobiological processes towards pathogenesis, neurodegeneration and clinical symptoms in people with AD. This knowledge is central to moving the field towards an accurate and personalized diagnosis of AD but also for the successful treatment of the disease. The reviewed findings encourage the design of modern therapies of a combined type. One can envision that the emerging anti‐amyloid therapies will yield higher therapeutic effects if combined with interventions targeting the neuroinflammation, brain iron, cholinergic dysfunction and cellular senescence in people with AD. However, other pathways and mechanisms not reviewed here may also be relevant for curing AD, and preventive lifestyles should not be discarded for the overall reduction of risk of AD.

## Conflict of interest statement

**Daniel Ferreira** consults for BioArctic and has received honoraria from Esteve Pharmaceuticals S.A. **Maria Eriksdotter** has served as a consultant for BioArctic AB, Roche, Eli Lilly, Biogen/Eisai and Novo Nordisk and given lectures in symposia sponsored by Roche and Bioarctic/Eisai. All other authors declare no conflicts of interest.
